# Complete Genome Sequence of Glutamicibacter mysorens NBNZ-009, Isolated from Jin Lake Sediment

**DOI:** 10.1128/mra.00762-22

**Published:** 2022-09-07

**Authors:** Yueying Wang, Xin Su, Jie Liu, Wei Fang, Lei Zhu

**Affiliations:** a National Biopesticide Engineering Technology Research Centre, Hubei Biopesticide Engineering Research Centre, Hubei Academy of Agricultural Sciences, Biopesticide Branch of Hubei Innovation Centre of Agricultural Science and Technology, Wuhan, People’s Republic of China; University of Southern California

## Abstract

Members of the genus *Glutamicibacter* have been reported from soil samples or some industrial pollution environments. Here, we present the genome of strain Glutamicibacter mysorens NBNZ-009, which was isolated from sediment from Jin Lake (Wuhan, China). The genome consists of a 3.68-Mbp circular chromosome and possesses 3,372 coding sequences.

## ANNOUNCEMENT

Recently, some species of the genus *Arthrobacter* were reclassified into a novel genus, *Glutamicibacter* gen. nov. (1). Members of *Glutamicibacter* have been isolated from soil samples or some industrial pollution environments (2). This study aimed to isolate ammonia-degrading bacteria from sediments. Glutamicibacter mysorens strain NBNZ-009 was isolated from samples that had been collected at a depth of 0 to 20 cm below the shallow sedimentary layer of Jin Lake (Wuhan, China [114.1917°N, 30.6516°E]) in 2021. A total of 25 distributed samples were collected (1 to 2 kg each). One-gram sediment samples were serially diluted with sterile water and were spread on lysogeny broth (LB) plates. After incubation for 2 days at 30°C, a single colony was purified by repeated streaking on LB plates. To characterize the strain, the 16S rRNA gene sequence was obtained by PCR amplification with the universal primers 27F and 1492R (3). Phylogenetic analysis was performed using the EZBioCloud database (4). NBNZ-009 showed the greatest similarity to the G. mysorens type strain LMG 16219 (GenBank accession number AJ639831.1) (99.52% identity).

Purified colonies were inoculated into LB medium and cultivated to the mid-logarithmic growth stage at 30°C. Genomic DNA for both long- and short-read sequencing was prepared using the blood and cell culture DNA kit (Qiagen, Germany) according to the manufacturer’s instructions. DNA concentrations were determined with a NanoDrop One UV-visible spectrophotometer (Thermo Fisher Scientific, USA) and a Qubit 4.0 fluorometer (Invitrogen, USA). Sample integrity and purity were determined by 0.75% agarose gel electrophoresis. For long-read sequencing, size selection was performed using the PippinHT system (Sage Science, USA). The DNA library was prepared using a ligation sequencing kit (SQK-LSK-109; Oxford Nanopore Technologies [ONT], Oxford, UK) without DNA shearing and was sequenced with a Nanopore PromethION sequencer (ONT) on an R9.4.1 flow cell (FLO-MIN106). Base calling, barcode segmentation, and removal of adapter sequences for the raw sequences were performed using Guppy v.4.4.2 (5). In this study, default parameters were used for all software. A total of 128,263 reads (3,014 Mb), with an average length of 23,504 bp, were generated; the *N*_50_ value was 30,457 bp. For short-read sequencing, genomic DNA was randomly fragmented with an LE220 focused ultrasonicator (Covaris, USA), and the DNA fragments were selected with AMPure XP magnetic beads (Beckman, Germany) to an average size of 200 to 400 bp. A paired-end DNA library was prepared following the MGIEasy FS PCR-free DNA library preparation set (catalog number 1000013455; MGI Tech Co., Ltd.). Sequencing was performed on an DNBSEQ-T7 instrument (MGI Tech Co.), using 150-nucleotide-long reads. Raw data were processed using the FASTQ preprocessing program fastp v.0.23.2 (6) for the purpose of trimming adapters and low-quality data, which yielded 7,986,830 short reads, with a total length of 1,197,917,560 bp.

The high-quality long- and short-read sequences were assembled into a complete and circularized chromosome using Unicycler v.0.4.8 (7). The average coverage is 1,144×. The chromosome of G. mysorens strain NBNZ-009 is 3,681,770 bp in length, with a GC content of 61.7%, and contains 3,372 coding sequences, 65 tRNA genes, 19 rRNA genes, and a CRISPR array, as determined by NCBI PGAP v.5.3 (8).

### Data availability.

The 16S rRNA gene has been deposited in GenBank under the accession number OP020885.1. The whole-genome shotgun project has been deposited in GenBank under the accession number CP099453.1. The raw sequencing data have been deposited in the Sequence Read Archive (SRA) under accession numbers SRR19970076 (MGI Tech Co.) and SRR19974005 (ONT).

## References

[1] Busse HJ. 2016. Review of the taxonomy of the genus *Arthrobacter*, emendation of the genus *Arthrobacter sensu lato*, proposal to reclassify selected species of the genus *Arthrobacter* in the novel genera *Glutamicibacter* gen. nov., *Paeniglutamicibacter* gen. nov., *Pseudoglutamicibacter* gen. nov., *Paenarthrobacter* gen. nov. and *Pseudarthrobacter* gen. nov., and emended description of *Arthrobacter roseus*. Int J Syst Evol Microbiol 66:9–37. doi:10.1099/ijsem.0.000702. 26486726

[2] Das L, Deb S, Das SK. 2020. *Glutamicibacter mishrai* sp. nov., isolated from the coral *Favia veroni* from Andaman Sea. Arch Microbiol 202:733–745. doi:10.1007/s00203-019-01783-0. 31796989

[3] Lane DJ. 1991. 16S/23S rRNA sequencing, p 115–175. *In* Stackebrandt E, Goodfellow M (ed), Nucleic acid techniques in bacterial systematics. John Wiley & Sons, New York, NY.

[4] Yoon SH, Ha SM, Kwon S, Lim J, Kim Y, Seo H, Chun J. 2017. Introducing EzBioCloud: a taxonomically united database of 16S rRNA gene sequences and whole-genome assemblies. Int J Syst Evol Microbiol 67:1613–1617. doi:10.1099/ijsem.0.001755. 28005526 PMC5563544

[5] Wick RR, Judd LM, Holt KE. 2019. Performance of neural network basecalling tools for Oxford Nanopore sequencing. Genome Biol 20:129. doi:10.1186/s13059-019-1727-y. 31234903 PMC6591954

[6] Chen S, Zhou Y, Chen Y, Gu J. 2018. fastp: an ultra-fast all-in-one FASTQ preprocessor. Bioinformatics 34:i884–i890. doi:10.1093/bioinformatics/bty560. 30423086 PMC6129281

[7] Wick RR, Judd LM, Gorrie CL, Holt KE. 2017. Unicycler: resolving bacterial genome assemblies from short and long sequencing reads. PLoS Comput Biol 13:e1005595. doi:10.1371/journal.pcbi.1005595. 28594827 PMC5481147

[8] Li W, O'Neill KR, Haft DH, DiCuccio M, Chetvernin V, Badretdin A, Coulouris G, Chitsaz F, Derbyshire MK, Durkin AS, Gonzales NR, Gwadz M, Lanczycki CJ, Song JS, Thanki N, Wang J, Yamashita RA, Yang M, Zheng C, Marchler-Bauer A, Thibaud-Nissen F. 2021. RefSeq: expanding the Prokaryotic Genome Annotation Pipeline reach with protein family model curation. Nucleic Acids Res 49:D1020–D1028. doi:10.1093/nar/gkaa1105. 33270901 PMC7779008

